# Notes and Comments

**Published:** 1921-12

**Authors:** 


### HOSPITAL and HEALTH REVIEW

### NOTES AND COMMENTS.

SIR ALFRED MOND stated, in the course of the debate on November 2 on the National Health Insurance (Prolongation of Insurance) Bill, that a Bill consolidating the Insurance Acts was in draft and would, he hoped, be introduced next year. This is good news; but we hope that Sir Alfred Mond is also going to undertake at an early date a far more important and necessary task—the consolidation of the Public Health Acts. The Acts in their present form (or want of form) are a disgrace to the Statute-book. The learned and capable editors of "Lumley" have done all that can be done to elucidate the law for us; but it is none the less deplorable that two immense volumes should be necessary for the purpose. Consolidation and simplification would be welcomed by every local authority and by every public-health authority in the country. It would make for economy as well as for efficiency, and there is no more useful work to which the Ministry of Health could apply itself, especially at a time when considerations of economy have compelled it to suspend many of its labours in other directions. The Board of Education have set a good example with their consolidating Bill this year (the Education Act, 1921), and we look to the Ministry to follow suit. There are other Acts much in need of consolidation, such as the Housing Acts; but the case of the Public Health Acts is urgent, and the work should be put in hand without delay.

IN this number we publish a short account of the outbreak of smallpox at Nottingham, and give our readers the second half of an interesting article on Edward Jenner. We print also in this issue a review and criticism, by Dr. C. Killick Millard, of the Memorandum on Vaccination and Smallpox which was recently published by the Ministry of Health. Those of our readers who have taken an interest in the article on Jenner will have read that anti-vaccinators flourished side by side with Jenner's great discovery, and that they attributed to vaccination even more ridiculous calamities than are ascribed to it by the anti-vaccinators of to-day. Dr. Millard, the Medical Officer of Health of Leicester, which is a town where vaccination is not practised, is a firm believer in the efficiency of vaccination to protect the individual against smallpox. Time and again smallpox has been introduced into Leicester, but by the diligent following up, isolation, and vaccination of contacts, no large epidemic has occurred in that town. So, regarding this article by Dr. Millard, although he questions some of the generally held statements regarding the value of the vaccination of children, it must not be supposed that he belongs to the cranky crowd of anti-vaccinators. There is certainly room for two opinions as to whether the partial vaccination of the population, as it is practised to-day, is beneficial to the public health, but, unfortunately, on this vexed subject of vaccination it is difficult to obtain unbiassed scientific opinion. Most of us, as Dr. Millard points out, are brought up from our earliest medical days in a strong pro-vaccinist atmosphere; and, for our own part, we have observed that our opinion in favour of vaccination is intensified every time that we meet an anti-vaccinist or read anti-vaccination pamphlets. Bernard Shaw, in the third act of "The Doctor's Dilemma," makes one of his characters say: "There are some things that place a man socially; and anti-vaccination is one of them." We should like to see the whole question of vaccination against smallpox discussed academically and without animus, especially as there is reason to think that the type of smallpox (like that of scarlet fever) is altering, and that the disease is now less virulent, and perhaps less infectious, than in former years. In epidemiological circles interest has been lately aroused by the disease called variously alastrim, Kaffir-pox, or varioloid varicella; and it would seem, as the result of recent laboratory work in America, that alastrim and smallpox are essentially identical. The authors of this American work note in their report that three facts about vaccination and smallpox are not generally recognised; first, that there is an acceleration of the reaction to vaccination in

**NOTES AND COMMENTS—(*cont.*).**

immune individuals; secondly, there is a frequent absence of complete immunity to vaccine virus after an attack of smallpox; and, thirdly, that there is an occasional absence of immunity to smallpox after successful vaccination or after an attack of smallpox itself. We do not believe that the last word has yet been said regarding the relation of smallpox and vaccination, or about the variation in the types and manifestation of the disease; and we should therefore welcome impartial research and discussion on these subjects.

---

THE Annual Report of the Chief Medical Officer of the Board of Education for the year 1920 has recently been published, and can be obtained from H.M. Stationery Office or from any bookseller for the sum of six shillings. This latest report by Sir George Newman is a document of profound sociological importance, and, like the previous reports upon the school medical service, it is yet one other milestone which marks the way of progress. Fourteen years have passed since the State first became interested in the health of the rising generation; and despite the years of war from which we have suffered, it is obvious, on reading this report, that our school medical services throughout the country have made, and are making, steady progress in the amelioration of those conditions which cripple and incapacitate the parent and wage-earner of the future. We all of us know what a large amount of invalidism exists among adults. During the war the Ministry of National Service told us that out of every ten men of military age in the country only three were perfectly fit and healthy, and we have been told also how year by year in England over 270,000 years of work are lost on account of sickness. It is to prevent this ill-health, this crippling and disability, that the school medical service has been working; and already there are some results—some signs of improved health in the children. In another generation, when the children whose health we are preserving to-day themselves have become parents, we may hope to find still further reward for our labour. In the meantime, we need especially hope, resolution, and patience to carry us over the lean years with which we are faced; and we may be certain, amid the difficulties and troubles with which we are surrounded to-day, that the end is within sight, and that, despite discouragement and bitterness, we are working for the welfare of mankind.

---

SOME of the doctors in Willesden seem to have a complaint against the Willesden District Council, apparently because of the completeness of the medical services of that authority. In Willesden, as in other towns in this country, the local authority supervises child life from birth or before birth until the child leaves school. The minor ailments of the children are prevented from becoming serious by early and adequate treatment, and no humanitarian would object to that. The care of child life was in the hands of nobody in particular until twelve years or so ago, when the State assumed the responsibility; and, in view of the vast amount of invalidism among adults to-day, it is doubtful if the older method of not caring for the children was altogether satisfactory in its results. The family doctor, greatly to his credit, does not, in the majority of towns, tout for fees or for patients; but the Health Authority, having statutory obligations to fulfil, is in a different position—it is part of the duty of the Health Authority to insist, for example, that its ailing school children are properly treated; and so it encourages them to come to the clinics and elsewhere for the remedy of minor ailments.

---

THIS grumble by the Willesden doctors has found its way, unfortunately we think, to the public Press. We question if these doctors have any real cause for complaint because the Willesden Council has made provision for the treatment of babies and school children. No doubt the doctors are losing a few small fees from these non-panel patients; but against that is the large advantage that they will reap later when these children of to-day become the panel patients of to-morrow. Our maternity and child-welfare schemes and our school medical services throughout the country are making sickly children into healthy adolescents, such as any doctor would welcome on his panel. Nowadays a child leaves school with no crippling conditions. It is a healthy child, and will not be in and out of the panel doctor's consulting-room half the year. This work of early, and, therefore, of successful, treatment is just the work which will render the life of the panel practitioner more and more easy with the passing years.

---

WE hope that our readers and others will give liberally this Christmas to the hospitals, which more than ever are in need of help at present. Christmas in hospital, and especially in a children's hospital, is a very jolly time, and it would be tragic if from lack of money or presents any hospital were forced to abandon its Christmas-trees. In giving liberally to the hospitals, as the public has always done at this season of the year, there has been a tendency to forget the isolation or fever hospitals, and in some of the smaller of these the cost of delighting the children at Christmas has fallen often enough upon the doctors and nurses of the institutions. Isolation hospitals are largely peopled by patients who are children, and they, much more than the grown-ups, look forward to the magic and glitter of Christmas-time.

---

AN admirable method of aiding the finances of the hospitals is suggested in a recent issue of "Country Life." Our contemporary writes: "One cannot help thinking that the time has come for the people of this country to lay aside the little bit of Pharisaism with which they regard racing. Let racing be acknowledged as a legitimate amusement,

**NOTES AND COMMENTS—(*cont.*).**

and establish for its management some institution akin to the *Pari-mutuel* of France, and the hospital difficulty would be got over without expense to the taxpayer. The essence of the *Pari-mutuel* is that a small, but not unimportant, proportion of the income goes to the Government. It is not desirable that they should treat this as though it were part of the ordinary revenue of the country. They could earmark it for the hospitals, and, seeing the popularity of racing in this country and the amount of betting that goes on, it is more than possible that the whole of the subsidiary funds required for making the hospitals self-supporting could be obtained from that source." We welcome this suggestion, which would not only aid the hospitals, but add also to the dignity of racing.

---

A SEVERE type of diphtheria has recently been prevalent in Bristol, and the medical officer of health, Dr. D. S. Davies, has introduced into certain selected institutions in the city the actrine immunisation, following Schick, which, as our readers may remember, is largely practised in America. One school has already been dealt with, and the medical officer of health proposes to continue this immunisation work in other schools. In addition, Dr. Davies and the resident medical officer of the Isolation Hospital have sent to practitioners in Bristol the following circular regarding the virulent type of diphtheria that prevails in the city:—

Certain virulent cases of diphtheria occurring in the city present signs which depart from those usually found in the disease, and render the diagnosis obscure in the early stages. These characters are a rapidly forming and intense œdema of the fauces, often with a peculiar translucent or gelatinous appearance of the uvula, with little or no reddening of the palate; a soft, almost painless œdema of the neck, extending from the cervical glands, and a much delayed formation of membrane. In some of the most intensely virulent cases little or no membrane may form for two or three days, by which time the patient is too much intoxicated to recover even with large doses of antitoxin. In other cases the exudate may be of a soft friable description superimposed on an intensely swollen tonsil. Some of these cases may be mistaken for quinsy, but may be distinguished from this disease by the œdematous appearance of the tonsil contrasted with the deeply engorged and usually unilateral swelling in quinsy, which pushes the soft palate downwards and forwards. In quinsy also the palate usually is reddened, while in diphtheria the palate is usually little altered in colour. The hard, painful cervical gland on the affected side in quinsy differs from the characteristic soft, almost painless œdema of the neck in this type of diphtheria. Some very severe cases recently have affected principally the naso-pharynx, with but slight involvement of the fauces. All these cases show is a thick purulent or sanious discharge from the mouth and back of the throat, sometimes with œdema of the nose and eyelids. The characteristic fœtor of the breath is a valuable sign, rarely absent in cases of diphtheria affecting either fauces or naso-pharynx, but not evident as a rule when confined to the larynx. The virulent cases require a dose of 20,000 units, even if seen on the first day, preferably given intramuscularly into the vastus externus muscle. If the œdema goes on increasing this dose should be repeated within not more than twenty-four hours. A smaller dose than this is insufficient to check this type of case. To postpone the administration of antitoxin while awaiting the result of a culture is fatal. Few of this type of case survive if the administration of antitoxin is delayed beyond forty-eight hours. As this type of throat does not seem to be very painful, children may only complain of headache or sickness, so it is advisable not to overlook the examination of the throat, even if the symptoms are not referred to it. Pyrexia is usually present on the first two days, but may be slight. After this interval the temperature may become normal or subnormal, with increasing intoxication of the patient. The possibility of conjunctival or vulvar cases should not be forgotten, as a fatal case of each has been admitted into this hospital during the past month, with intense hæmorrhagic œdema of the infected area.

---

WE understand that Bradford, Leeds, Cardiff, and Burton are now included in the centres which have decided not to join the scheme for the appointment of Local Committees, advocated by the Report of the Cave Committee, and now in process of formation under the Voluntary Hospitals Commission, and with the approval of the Ministry of Health. With the point of view of these and other abstainers we have dealt carefully in each of our previous recent issues. It is encouraging to note that the arguments set forth therein remain unanswered. If they continue to remain so we shall be forced to conclude that the arguments are also unanswerable.

---

IT is well that attention has been called in good time to the form in which each individual entry against a registered nurse's name shall appear in the State Registers now being compiled. The Rules made under the English Act are clear on the point; Rule 3 (3) limits the particulars which the Register shall contain to those set forth in the first schedule —viz. name, address, date of registration, qualification—i.e. whether the nurse is qualified as an "existing nurse," or a "nurse with intermediate qualification," or a "nurse by examination," and in what hospital or other institution she has received training, and date. It is of interest in this connection to note that the Scottish Council has resolved that its Register shall contain under "Qualification" the letters "E.N." (Existing Nurse) or "N.T." (Nurse in Training) only. Further, that in the case of such nurses with a complete three years' training, the columns "Date of obtaining qualification" and "Hospitals in which qualifying training received" should be filled up, while in the case of such nurses not having a complete three years' training in a recognised hospital, the columns should be left blank.

---

AN interesting experiment which should help to increase the supply of nurses in the United States of America is being made by the United States Public Health Service. A training-school, the headquarters of which are at the Surgeon-General's

NOTES AND COMMENTS—(*cont.*).

Office at Washington, has been established, which offers to women desiring to take up the profession of nursing a course of study leading to a diploma, and an opportunity to assist in caring for disabled military patients. Candidates must attain a high school standard, and preference is given to those who have taken the course in elementary hygiene and home care of the sick with the Red Cross or who served as nurses and aides in Army and civilian hospitals throughout the war. Training will be given in certain hospitals of the Public Health Service, which provide experience in medical, surgical, special, and public-health nursing. The course is three years, but to graduates and students of accredited colleges certain allowances in time are made. No tuition fee is required, and students are provided with quarters, subsistence, laundry, and text-books, whilst they receive thirty dollars a month for the first two years and fifty dollars a month for the third year towards the cost of their uniform and other expenses.

---

ARISING out of the Cave Report much work of a new kind has fallen on King Edward's Hospital Fund. This, with the increase in the volume of the old work due to post-war conditions, has necessitated reorganisation and the reconstitution of the committees; a new Management Committee has been formed consisting of the Chairmen of the other committees, which by meeting often will be able to deal quickly with emergency matters. At a special meeting of the King's Fund held on November 9 the Chairman of the Executive Committee (Lord Stuart) stated that the measures for the preservation of the voluntary system are being worked out in consultation with the hospitals, and no final report is yet possible. At the same meeting a resolution was passed expressing the hope that the pound-for-pound condition attached by the Treasury to the temporary assistance voted by Parliament to voluntary hospitals will not be pressed. The Executive Committee is of opinion that the application of this condition, which was not recommended in the report of Lord Cave's Committee, nor separately considered (much less expressly enjoined) by Parliament, will lead to great difficulties, and probably to great injustices as between different hospitals in the distribution of the grants.

---

AT the meeting of the General Medical Council held on November 22, Sir Donald MacAlister, the President, made an announcement regarding the policy of the Public Health Committee of the Council in respect to the training for Diplomas and Degrees in Public Health. Under the existing regulations, a doctor who wishes to take a public health qualification has to pursue a nine months' course of study. This course is chiefly academic, in that it consists far more in attending lectures and in working in a laboratory than in active and practical work in a public health department. It is true that a student is supposed to do a course of outdoor work under a medical officer of health; but this "outdoor work" consists in inspecting a few nuisances, slaughter-houses and common lodging-houses, and does not really bring the student into close touch with the realities of public health administration. The result of this inadequate instruction is that the student is not fully proficient in all the branches of knowledge of State Medicine. In the words of Sir Donald MacAlister, "It would appear that for the modern Public Health service, the qualification to be required before admission to office should be more than an academic certificate, attainable after nine months or so of additional study, by a licentiate or graduate who has just attained his first licence to practice. It should imply a greater maturity of outlook, a deeper knowledge of principles, and a fuller practical acquaintance with actual administrative methods than an inexperienced practitioner can fairly be expected to compass in a year. Something equivalent to a short apprenticeship in a working Public Health department is, in fact, needed to supplement the lectures and demonstrations in the class-room and laboratory." We agree entirely with this, and hope that these wise observations will before long be expressed in regulations that will enlarge the present course of study of candidates for public health qualifications.

---

BUT it is not only the student for a public health diploma who needs to receive better instruction in preventive medicine. If the general practitioner of the future is to take any useful part in the prevention of disease, he requires to be more fully instructed while he is yet a medical student. To-day a newly qualified medical student goes out to practise medicine armed with a little midwifery, a fair amount of surgery and medicine, and a vast belief in the value of the pharmacopœia. He may know how to fill in a death certificate, and may have some idea of his duties regarding the notification of infectious diseases; but he knows little or nothing about infant hygiene, the school medical services, or the vast possibilities of preventive medicine. He inclines to be a little plaintive about the public health services, because he thinks they are intending to rob the family doctor. If every medical student received instruction in the principles and practice of preventive medicine, this vague hostility would be changed to a real enthusiasm; and in a generation we should see an immense improvement in the health of the people and a lightening of the burden of the panel doctor's existence.

---

IT has been held in the House of Lords that the Glasgow Corporation is liable for damages on account of the death of a child from eating belladonna berries plucked in its Botanic Garden. "A Ratepayer" suggests that if this precedent is adopted, those who are responsible for our open spaces will be under the necessity of taking absurd precautions to safeguard the lives and limbs of their "young visiters." A better way, we think, would be to get a short Act passed, conferring summary spanking powers on park-keepers.

